# Highlight report: Role of choline phospholipid metabolism in tumor progression

**DOI:** 10.17179/excli2019-2071

**Published:** 2019-12-19

**Authors:** Wiebke Albrecht

**Affiliations:** 1 Leibniz Research Centre for Working Environment and Human Factors

## ⁯⁯⁯⁯

Recently, Sonkar and colleagues published a comprehensive review about the glycerophosphocholine (GPC) pathway and its role in cancer biology (Sonkar et al., 2019[24]). The authors address the enzymes of the GPC breakdown pathway; the oncogenic signaling pathways and transcription factors that regulate the GPC pathway; the interactions of the GPC pathway with other biochemical pathways such as glycolysis and triglyceride metabolism, and finally focus on non-invasive magnetic resonance spectroscopy techniques for detection of GPC in tumor tissue. Activation of choline metabolism is a critical step of cancer development and may lead to increased levels of phosphocholine, GPC and total choline-containing compounds (Hanahan and Weinberg, 2011[14]; Griffiths et al., 1981[13]; Daly et al., 1987[8]; Aboagye and Bhujwalla, 1999[1]; Gillies et al., 2002[10]). These changes can be detected by magnetic resonance spectroscopy (Baek et al., 2008[3]; Bolan et al., 2003[4]; He et al., 2003-2004[15]; Al-Saffar et al., 2017[2]; Glunde et al., 2011[11]). The glycerophosphodiesterase GPCPD1 is a key enzyme in choline metabolism that cleaves GPC to glycerol-3-phosphate (G3P) and choline (Stewart et al., 2012[25]). This activity mediates integrin expression, tumor cell adhesion, spreading and migration (Lesjak et al., 2014[17]; Marchan et al., 2012[19]). Recently, glycerol-3-phosphate acyltransferase1, which further processes G3P to generate lysophosphatidic acid (LPA) has been shown to promote tumor cell migration and is associated with poor survival in ovarian cancer (Marchan et al., 2017[19]). 

Besides phospholipid metabolism and metastasis tumor progression involves numerous further processes, such as proliferation (Schmidt et al., 2008[20]; Hellwig et al., 2016[16]; Siggelkow et al., 2012[23]), immune cell infiltration (Edlund et al., 2019[9]; Schmidt et al., 2012[21], 2018[22]; Godoy et al., 2014[12]), oxidative stress response (Cadenas et al., 2019[6]; 2014[5]; 2012[7]). Studies in future will have to show if targeting members of the glycerophosphocholine pathway will delay tumor progression. The present review of Sonkar et al. gives a comprehensive summary of the state-of-the-art in this field and is of high value to anyone interested in how choline-phospholipid-metabolism is linked to tumor development. 

## Conflict of interest

The author declares no conflict of interest.
